# Supplementary material for: Chromothripsis during telomere crisis is independent of NHEJ, and consistent with a replicative origin
Source: Genome Res. 2019 May;29(5):737–49. doi: 10.1101/gr.240705.118 (PMC6499312; doi:10.1101/gr.240705.118)
Supplement: Supplemental Material [file supp_gr.240705.118_Supplemental_file_1.zip › contigs/annotated_contigs/DB116/contig.2.DB116_length_573_mean_cov_9.19371727749.docx]

**DB116_length_573_mean_cov_9.19371727749**

```
GAGGTCAGGAGATCGAGACCATCCTGGCTAACACGGTGAAACCCCGTCTCTACTAAAAATACAAAAAATTAGCCGGGCGCGGTGGCGGG
 >chr2:104759203-104759437 + E=4e-120 p=1e-02
CGCCTGTAGTCCCAGCTACTTGGGAGGCTGAGGCAGGAGAATGGCGTGAACCCGGGAAGCGGAGCTTGCAGTGAGCCGAGATCCCGCCA

CTGCACTCCAGCCTGGGCGACAGAGCGAGACTCCGTCTCAAAAAAAAAAAAA|AAAA|TTTCATGAACAATATCTTAGATATTTCTTAG
                                                     >chr1:100994210-100994555 + E=6e-1
TTATGTTGTGATTTTGTCAATGCTGCTTAACTCTAATGCAGGATGATAATTGTGGCTAATTGCCTAAAAGCACATGAACTTTTTTTCCC
87
ATCAGTGTTATATGTGAGAGAACACACACACACACACACACACACCCCTCTTTAATATGAATTTGATGTCCTCACTTAAATTACATGAA

ATAATTCCTAAATGCTTGAAAATACAAAACCCTCCTCATCTAGTTCCTATCTCAATCAGACAAATATTTTAAAGCAGATTCAAAAAATA

GACTATACTAAACTTCTTACTGGAAAAAAAGTCACTTCTGG
```
